# Staffing Patterns in US Nursing Homes During COVID-19 Outbreaks

**DOI:** 10.1001/jamahealthforum.2022.2151

**Published:** 2022-07-22

**Authors:** Karen Shen, Brian E. McGarry, David C. Grabowski, Jonathan Gruber, Ashvin D. Gandhi

**Affiliations:** 1 Department of Health Policy and Management, Bloomberg School of Public Health, Johns Hopkins University, Baltimore, Maryland; 2 Division of Geriatrics & Aging, Department of Medicine, University of Rochester Medical Center, Rochester, New York; 3 Department of Health Care Policy, Harvard Medical School, Boston, Massachusetts; 4 Department of Economics, Massachusetts Institute of Technology, Cambridge; 5 UCLA Anderson School of Management, University of California, Los Angeles

## Abstract

**Importance:**

Staff absences and departures at nursing homes may put residents at risk and present operational challenges.

**Objective:**

To quantify changes in nursing home facility staffing during and after a severe COVID-19 outbreak.

**Design, Setting, and Participants:**

In this cohort study, daily staffing payroll data were used to construct weekly measures of facility staffing, absences, departures, and use of overtime and contract staff among US nursing homes experiencing a severe COVID-19 outbreak that started between June 14, 2020, and January 1, 2021. Facility outbreaks were identified using COVID-19 case data. An event-study design with facility and week fixed effects was used to investigate the association of severe outbreaks with staffing measures.

**Exposures:**

Weeks since the beginning of a severe COVID-19 outbreak (4 weeks prior to 16 weeks after).

**Main Outcomes and Measures:**

Total weekly staffing hours, staff counts, staff absences, departures, new hires, overtime and contract staff hours measured for all nursing staff and separately by staff type (registered nurses, licensed practical nurses, certified nursing assistants), facility self-reported staff shortages, and resident deaths.

**Results:**

Of the included 2967 nursing homes experiencing severe COVID-19 outbreaks, severe outbreaks were associated with a statistically significant drop in nursing staffing levels owing to elevated absences and departures. Four weeks after an outbreak’s start, around when average new cases peaked, staffing hours were 2.6% (95% CI, 2.1%-3.2%) of the mean below preoutbreak levels, despite facilities taking substantial measures to bolster staffing through increased hiring and the use of contract staff and overtime. Because these measures were mostly temporary, staffing declined further in later weeks; 16 weeks after an outbreak’s start, staffing hours were 5.5% (95% CI, 4.5%-6.5%) of the mean below preoutbreak levels. Staffing declines were greatest among certified nursing assistants, primarily owing to smaller increases in new hires of this staff type compared with licensed practical nurses and registered nurses.

**Conclusions and Relevance:**

In this cohort study of nursing homes experiencing severe COVID-19 outbreaks, facilities experienced considerable staffing challenges during and after outbreaks. These results suggest the need for policy action to ensure facilities’ abilities to maintain adequate staffing levels during and after infectious disease outbreaks.

## Key Points

**Question:**

What is the association between severe COVID-19 outbreaks and US nursing home staffing patterns?

**Findings:**

In this cohort study of 2967 nursing homes in 2020, owing to increased absences and departures, there were statistically significant declines in staffing levels during a severe COVID-19 outbreak that remained statistically significantly reduced 16 weeks after the outbreak’s start. Facilities temporarily increased hiring, contract staff, and overtime to bolster staffing during outbreaks, but these measures did not fully replace lost staff, particularly certified nursing assistants.

**Meaning:**

Considerable staffing challenges suggest a potential need for policy action to ensure adequate staffing levels during nursing home outbreaks to protect resident health.

## Teaser

This cohort study quantifies changes in nursing home facility staffing during and after a severe COVID-19 outbreak.

## Introduction

Nursing homes have been an epicenter of the COVID-19 pandemic, and the tragic consequences for residents—including nearly 170 000 resident deaths as of May 2022 and months of extreme social isolation owing to visitor restrictions and reduced communal activities—have been well documented.^1,2,3^ Less, however, is known about the effects of the pandemic on the staff charged with caring for this vulnerable population.

The nursing home industry has long been plagued by concerns about the adequacy and turnover of its staffing,^4,5,6,7^ and anecdotal reports indicate that COVID-19 has placed immense additional strain on the industry’s workforce.^8^ Staff have been at substantial risk of contracting COVID-19, in part owing to persistent personal protective equipment shortages and inadequate COVID-19 testing.^9,10^ There were more than 1 million confirmed COVID-19 cases and more than 2300 deaths among nursing home staff as of early May 2022.^2^ The latter implies an annual death rate surpassing those of the most deadly occupations in the US, including commercial fishing and logging.^11,12^ Reports suggest that awareness of this risk has led some staff to leave their positions.^13,14^ When staff leave or are even temporarily absent owing to sickness or quarantine, facilities must find ways to stretch existing staff or bring on new workers to provide even basic care to residents.^8^

Despite these anecdotal reports, few empirical studies have documented the effect of COVID-19 on nursing home staffing. Existing studies have established a large decline in industry-wide employment, which was 13.2% lower in June 2021 than at the pandemic’s start.^15^ However, some researchers have also noted that because occupancy levels have also decreased considerably, staff hours per patient day (a common measure of staffing) have not generally decreased.^16^ Nevertheless, many facility managers have reported experiencing staffing shortages in survey data,^10^ suggesting that they may not feel that typical hours of care per patient are adequate given the challenges of providing care during the pandemic.

This cohort study uses daily individual-level staffing data and an event-study design to quantify the association between severe COVID-19 outbreaks and changes in nursing home staffing. The main research questions were (1) whether severe outbreaks were associated with declines in staffing and by how much; (2) whether these declines were primarily driven by temporary absences, permanent departures, or a lack of new hiring; (3) how facilities may have used overtime or contract staffing to cope with these declines; and (4) how these effects may have varied across the 3 primary nursing staff types: registered nurses (RNs), licensed practical nurses (LPNs), and certified nursing assistants (CNAs). To the extent that CNAs are paid lower wages and may perform tasks with the highest exposure risk, outbreaks may particularly affect their likelihood of being absent or leaving their jobs, as well as facilities’ abilities to fill these positions. On the other hand, RN and LPN positions could be more affected because as more highly certified and higher-earning workers, they may be most willing or able to leave their jobs.

## Methods

### Data

The primary data for this study are from the Centers for Medicare & Medicaid Services’ Payroll Based Journal (PBJ) system. Nursing homes are required to submit daily employee-level staffing information based on auditable payroll and contract data to the PBJ, including unique employee identifiers (allowing individual staff members to be tracked over time), staff type, employment arrangement (salary or contract), and hours worked.^6,7,16^ While the data cover a broad range of staff types, we restricted this analysis to nursing staff (RNs, LPNs, and CNAs) because these staff provide the majority of direct care for patients, and facilities may have deliberately reduced or deferred the use of other staff (eg, therapists, administrative staff) during outbreaks. These staff care for both short-stay and long-stay patients at nursing homes. We used data from January 1, 2017, through June 31, 2021, which covered an average of 333 million daily shifts for 3.6 million staff members at 15 518 facilities each year.

We obtained data on weekly resident and staff COVID-19 cases, resident COVID-19–related deaths and total deaths, and self-reported staff shortages reported from the National Healthcare Safety Network (NHSN) COVID-19 Nursing Home Data set.^2^ These data have been published weekly since May 24, 2020.

Per Harvard institutional review board policy, institutional review board approval and written informed consent were not required because this study uses publicly available data. This study follows the Strengthening the Reporting of Observational Studies in Epidemiology (STROBE) reporting guidelines for cross-sectional studies.

### Study Population and Exposure Measure

The population of interest was all US nursing homes, and the primary research objective was to quantify the association of severe COVID-19 outbreaks with changes in facility staffing. We defined outbreaks as contiguous weeks in which facilities reported new cases among residents or staff. We considered an outbreak to have ended after a 2-week stretch without new reported cases. We considered outbreaks that started between June 14, 2020 (the earliest possible date given available data), and January 1, 2021 (to focus on outbreaks prevaccination). We sorted these outbreaks by their severity, defined as total cases per bed, and defined the top decile of outbreaks across all nursing homes as severe outbreaks. Focusing on the top 10% of outbreaks ensured that we observed staffing patterns when facilities were most challenged and may have had the greatest difficulty maintaining adequate staffing levels.

The final analysis sample consisted of all facilities experiencing severe outbreaks that were able to be matched to the PBJ data. We kept only the first severe outbreak for each facility because later outbreaks may have been less comparable owing to spillover effects (increased immunity, staff departures) from the earlier severe outbreak (additional details in eAppendix in the Supplement). The key exposure measure was the weeks since each outbreak’s start.

### Staffing Measures

We constructed the following staffing measures at the facility-week level using the PBJ data.

#### Hires

We counted employees as hired in the first week they worked hours at a facility.

#### Absences

We labeled an employee as absent in any week between their first and last week of employment during which they did not log any hours. We capped the length of an absence at 12 weeks, meaning an employee who returned after not working for 13 or more weeks would be classified as a departure followed by a new hire. This ensures that absences are defined consistently throughout the sample.

#### Departures

We counted employees as departing a facility on the last week they logged hours at that facility before a period of at least 13 weeks without logging any additional hours at the facility.

#### Staff Count

We defined the staff count as the number of unique nursing staff who logged any hours at a facility in a given week.

#### Staff Hours

We calculated total weekly hours worked by nursing staff for each facility-week. We also separated these hours into regular-time hours (hours below 40 hours each week worked by noncontract employees), overtime hours (hours exceeding 40 hours each week worked by noncontract employees), and contract hours (all hours worked by contract employees).

### Other Measures

#### Staff Shortages

We defined a facility as reporting a staffing shortage in any week they reported a shortage of nursing staff or aides in the NHSN.

#### Resident Deaths (COVID-19 and Non–COVID-19 Related)

We used measures of resident deaths reported as due to COVID-19 and resident deaths not reported as due to COVID-19 from the NHSN data. Non-COVID-19–related deaths represent an extreme resident health outcome that could have been influenced by staff shortages.

### Statistical Analyses

We used an event-study framework to study how staffing patterns changed during and after a severe outbreak. We ran multivariable linear regressions where the unit of analysis was all available facility-weeks for facilities that experienced a severe outbreak. The primary exposure variables were event-time indicators defined as weeks relative to the start of the outbreak. Models also included facility and calendar-time fixed effects. The facility fixed effects ensure that the event-time indicators are identified from within-facility variation in staffing over time. The time fixed effects control for secular time trends affecting all facilities (eg, holidays, news about the pandemic). Thus, the specification is a 2-way fixed-effect event-study regression, where the identifying assumption is of parallel time trends (ie, that in the absence of an outbreak, facilities experiencing severe outbreaks would have had similar staffing patterns as facilities not currently experiencing severe outbreaks). We graphed the coefficient estimates on the event-time indicators between −4 and 16 weeks, along with their 95% CIs. The effects prior to the start of the outbreak serve as a test of the plausibility of the parallel trends assumption. We clustered standard errors by facility.

Using this method, we first explored how cases, total weekly nursing staff hours, resident census, and hours per resident evolved for the analysis sample during a severe outbreak. Next, we decomposed staffing changes into changes in absences, departures, new hires, overtime, and contract staffing, and studied the contribution of each of these components to the overall trend using the same event-study method. Next, we ran separate regressions for RNs, LPNs, and CNAs, and compared the average outbreak effect (the average coefficient across the first 16 weeks of an outbreak) across staff types. Finally, we plotted the event-study coefficients for self-reported staff shortages and resident deaths. Analyses were performed using Stata, version 16.1 (StataCorp).

### Sensitivity Analyses

In sensitivity analyses, we presented estimates for every outbreak decile to verify that effects were concentrated among larger outbreaks. We also redid the analysis defining severe outbreaks in terms of total cases, rather than total cases per bed. While this alternate definition is a more conventional way to define severe outbreaks, it naturally identifies larger facilities, which is why we used the per-bed definition in the main text. We also added controls for county-level case rates to provide reassurance that the observed associations are specific to a facility experiencing an outbreak, rather than a broader community-level outbreak. Finally, because the estimates exploit staggered event timing, we followed the literature and redid the analyses using alternative estimators that are robust to negative weighting.^17^

## Results

There were 39 390 COVID-19 outbreaks identified in the data with a mean of 29.6 resident and staff cases (0.30 cases per bed). The top decile of these outbreaks was labeled as severe outbreaks, which averaged 135.1 cases, corresponding to 1.5 cases per bed (eFigure 1 in the Supplement). Matching to the PBJ data and keeping the first severe outbreak for each facility resulted in a final analysis sample of 2967 nursing homes with severe outbreaks (additional details in the eAppendix and eTable in the Supplement).

**Figure 1.**
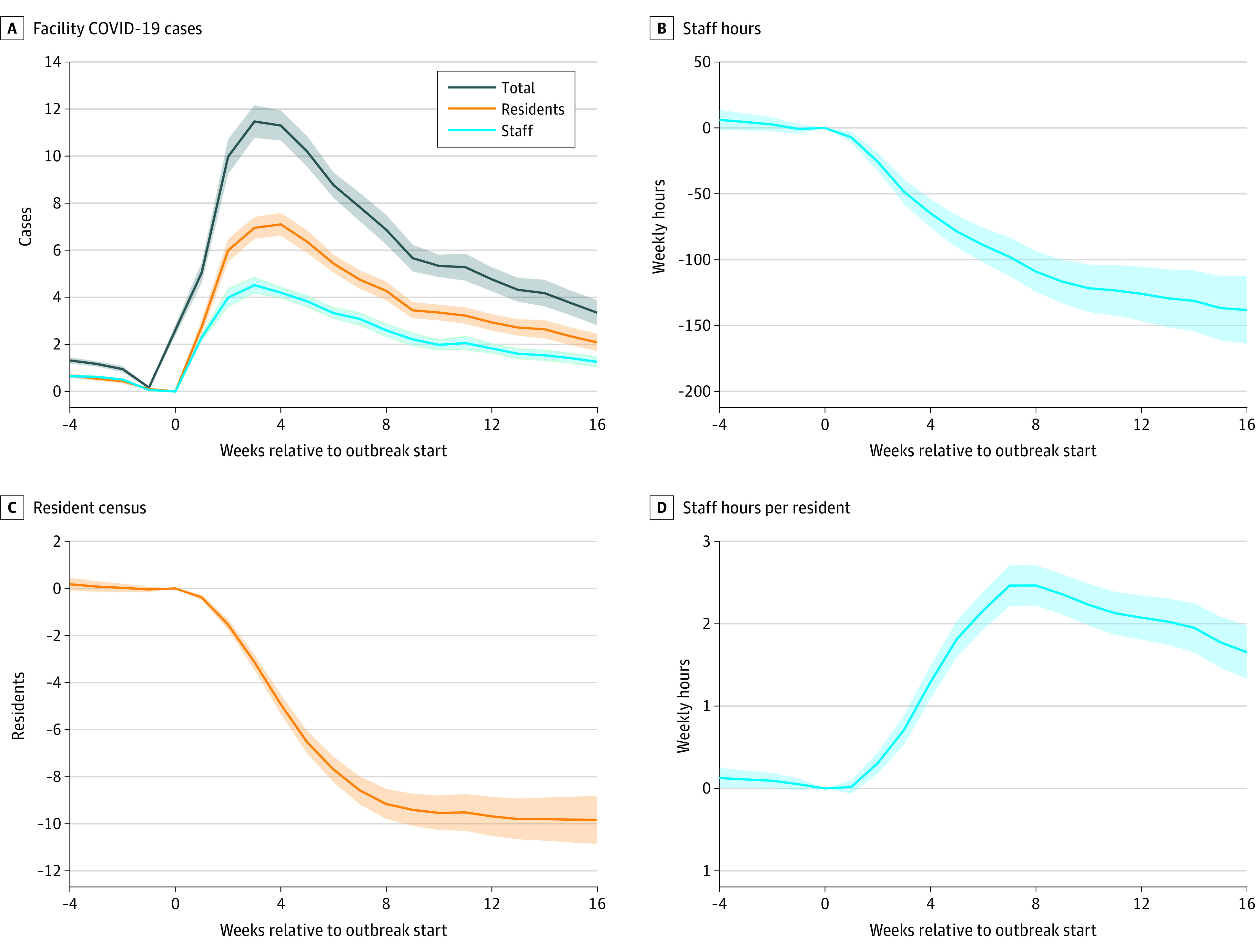
Nursing Home Staffing Measures and Resident Census During a Severe COVID-19 Outbreak Coefficients and 95% CIs (shaded areas) are shown from facility-week regressions for an analysis sample of 2967 facilities experiencing a severe COVID-19 outbreak that started between June 14, 2020, and January 1, 2021. There are 456 029 facility-weeks between January 1, 2017, and March 31, 2021, included. Primary independent variables are event-time indicator variables relative to the outbreak start. All regressions also contain facility and week fixed effects. Dependent variables are facility COVID-19 cases reported in the National Healthcare Safety Network data (A), total hours worked by nursing staff at a given facility in a given week from the Centers for Medicare & Medicaid Services’ Payroll Based Journal data (B), resident census in a given week from the National Healthcare Safety Network data (C), and nursing staff hours per resident (ie, the measure from panel B divided by the measure from panel C) (D).

Figure 1A shows that average weekly cases for the severe outbreak sample peaked at 11.5 cases in the third week and dropped to 3.3 cases in the 16th week. Figure 1B and C show that nursing staff hours and resident census dropped sharply at the start of severe outbreaks and continued to drop after the peak in average new cases. In week 4, hours of nursing staff care were down 65 (95% CI, 54-76) hours per week, and by week 16 they were down 138 (95% CI, 113-163) hours per week, a decline of 6.4% of the mean. Resident censuses also dropped precipitously during severe outbreaks to down 4.9 (95% CI, 4.5-5.3) residents after 4 weeks and 9.8 (95% CI, 8.8-10.9) residents after 16 weeks, or 12.4% of the mean decline. Because the decline in residents was steeper than the decline in staffing hours, Figure 1D shows that nursing hours per resident (a common measure of staffing) actually increased during severe outbreaks.

**Figure 2.**
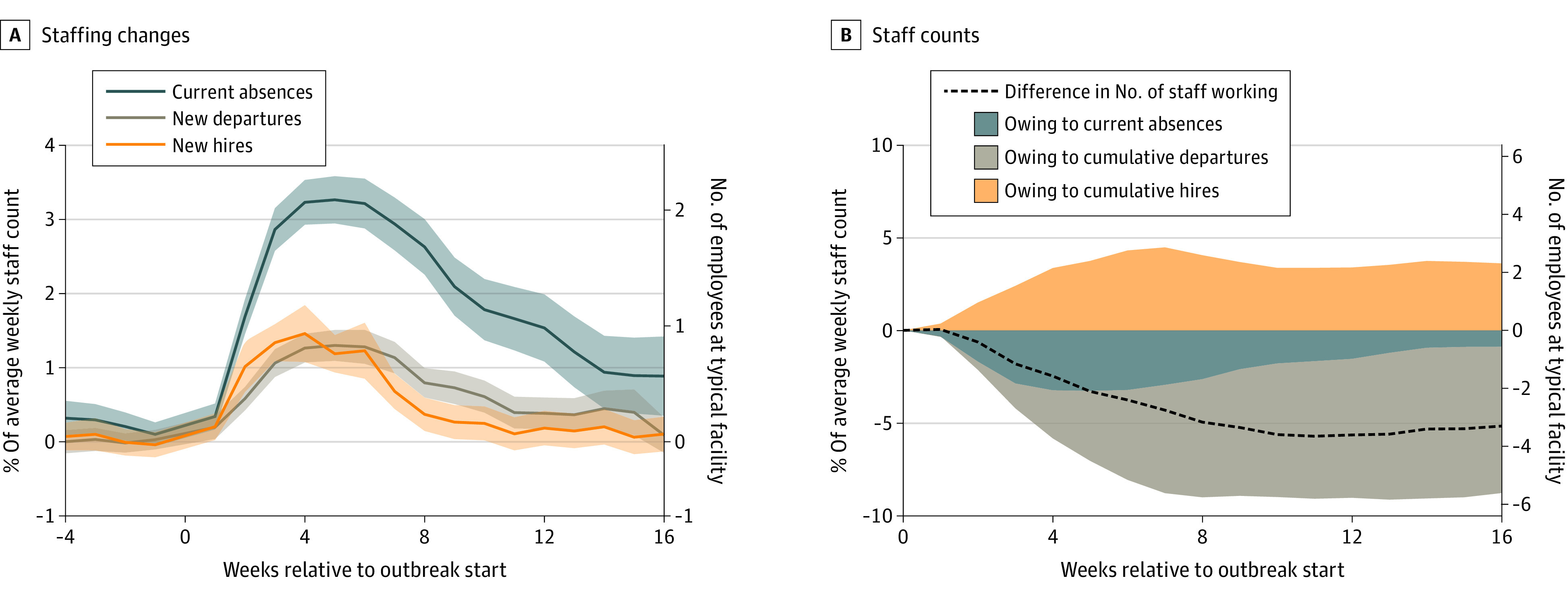
Additional Nursing Staff Absences, Departures, and New Hires During a Severe COVID-19 Outbreak and Association With Weekly Nursing Staffing Levels Coefficients and 95% CIs (shaded areas) are shown from facility-week regressions for an analysis sample of 2967 facilities experiencing a severe COVID-19 outbreak that started between June 14, 2020, and January 1, 2021. There are 456 029 facility-weeks between January 1, 2017, and March 31, 2021, included. Primary independent variables are event-time indicator variables relative to the outbreak start. All regressions also contain facility and week fixed effects. A, Dependent variables represent counts of nursing employees who were absent, departed, or hired in each week, scaled by each facility’s average weekly staffing level across all weeks. B, The dependent variable for the dashed line is the weekly nursing staffing level, the blue area uses current absences, the green area uses cumulative (past 12 weeks) departures, and the orange area uses cumulative (past 12 weeks) new hires, again all scaled by the average weekly staffing level. The right y-axis of each panel scales these effect sizes by the mean weekly staffing level across all facilities that experienced a severe outbreak.

Figure 2A shows that average weekly nursing staff absences, departures, and new hires all rose during the first few weeks of an outbreak and peaked around the same time as average cases. In week 4, an additional 3.2% (95% CI, 2.9%-3.5%) of the facility average staffing level was absent, an additional 1.3% (95% CI, 1.1%-1.5%) departed, and an additional 1.5% (95% CI, 1.1%-1.9%) were newly hired. The right y-axis rescales these estimates into counts for a typical facility with 64 nursing staff members working each week—this is equivalent to 2.0 additional absences, 0.8 departures, and 1.0 new hires. Absences remained elevated above normal levels 16 weeks after an outbreak’s start, while departures and new hires both returned closer to baseline levels. However, because departures consistently outnumbered new hires between weeks 5 and 15, the result was a net loss of employees during this time. Figure 2B graphs how these changes were associated with the facility’s staff count. While absences reduce the staff count only in the week that they occur, departures and new hires have a cumulative effect over time. Thus, in week 4, absences and cumulative departures each reduced the staff count by roughly the same amount. However, in week 16, the reduced staff count of 5.1% of the mean is largely owing to the cumulative effect of additional departures relative to new hires.

**Figure 3.**
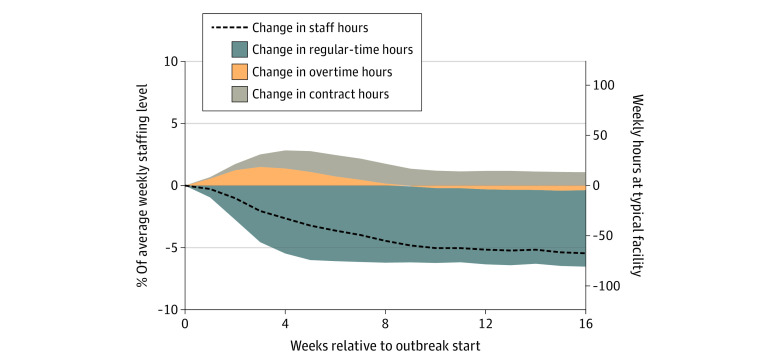
Use of Contract and Overtime Hours During a Severe COVID-19 Outbreak and Association With Weekly Nursing Home Staffing Hours Coefficient estimates and 95% CIs (shaded areas) are shown from facility-week regressions for an analysis sample of 2967 facilities experiencing a severe COVID-19 outbreak that started between June 14, 2020, and January 1, 2021. There are 456 029 facility-weeks between January 1, 2017, and March 31, 2021, that are included. Primary independent variables are event-time indicator variables relative to the outbreak start. All regressions also contain facility and week fixed effects. Dependent variables are weekly hours worked by nursing staff, scaled by each facility’s average across all weeks. The dependent variable for the dashed line is total weekly hours worked. The blue area uses regular-time hours (ie, hours worked by noncontract employees up to 40 hours per week), the orange area uses overtime hours (ie, hours worked by noncontract employees that are more than 40 hours in a week), and the gray area uses all hours worked by contract workers. Together, these areas sum to the dashed line shown. The right y-axis multiplies the effect size by the mean average nursing home weekly hours across all facilities that experienced a large outbreak.

Figure 3 explores 2 other ways facilities can try to cope with staffing losses: contract staffing and overtime. On average, regular-time hours dropped sharply during the first few weeks of an outbreak. Partly offsetting these declines, average overtime and contract hours both rose during the initial weeks of an outbreak. In week 4, though regular-time hours fell by 5.5% (95% CI, 5.0%-6.0%) of the mean, increased overtime and contract hours added an additional 1.3% (95% CI, 1.1%-1.5%) and 1.4% (95% CI, 1.1%-1.7%) of the mean to the staffing level, respectively. The net effect of these changes is that staffing hours were only reduced by 2.6% (95% CI, 2.1%-3.1%) in week 4. Consistent with these patterns, additional hiring was concentrated among contract employees (eFigure 2 in the Supplement). However, following the average peak weeks, the additional use of overtime and contract hours declined. Overtime returned to near-baseline levels around week 9, and additional contract hours declined to roughly 1% of the staffing level by week 10. Because regular-time hours did not recover, total staffing hours continued to decline past these peak weeks, reaching 5.5% (95% CI, 4.5%-6.5%) of the mean below preoutbreak levels by week 16.

**Figure 4.**
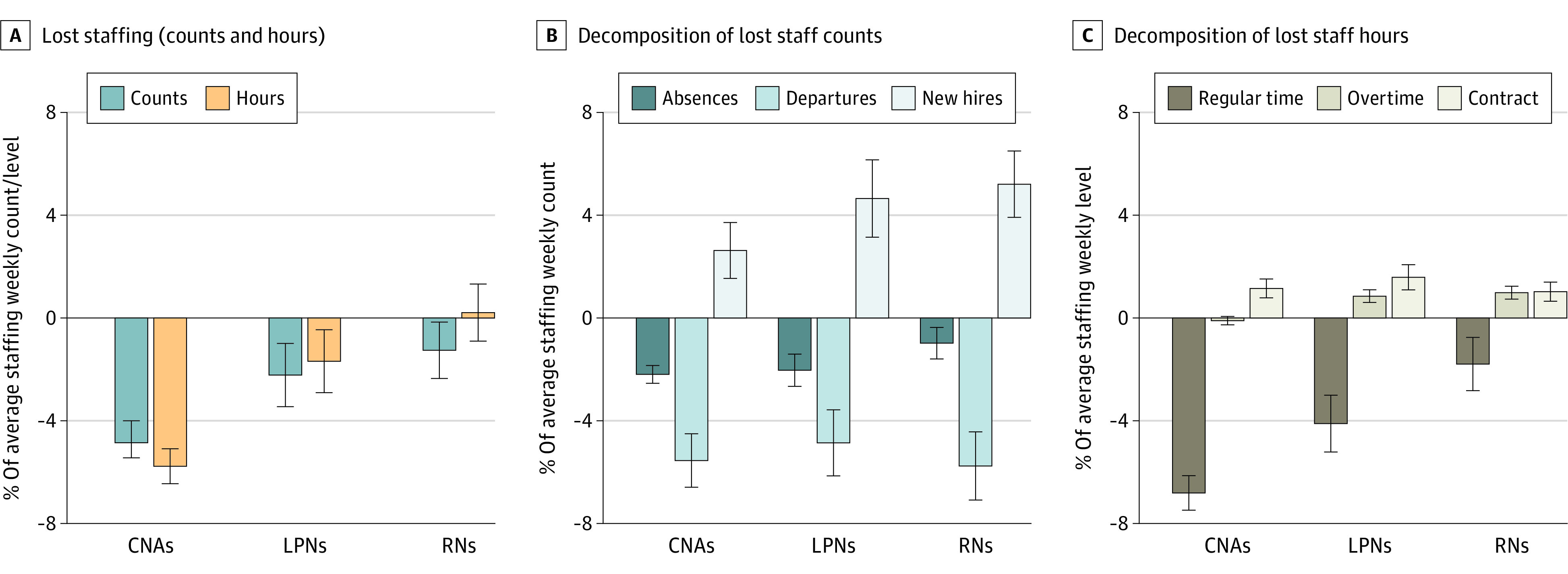
Summary of Average Weekly Change in Nursing Home Staffing Counts and Hours During a Severe COVID-19 Outbreak by Staff Type Coefficients and 95% CIs (error bars) are shown from facility-week regressions for an analysis sample of 2967 facilities experiencing a severe COVID-19 outbreak that started between June 14, 2020, and January 1, 2021. There are 456 029 facility-weeks between January 1, 2017, and March 31, 2021, included. Primary independent variables are event-time indicator variables relative to the outbreak start. Each bar represents the sum of these coefficients across the first 16 weeks after an outbreak’s start for a different dependent variable. A, The dependent variables are total staffing counts and hours for each of 3 staff types: registered nurses (RNs), licensed practical nurses (LPNs), and certified nursing assistants (CNAs). B, Dependent variables are absences, departures, and new hires for each staff type. C, Dependent variables are regular-time hours, overtime hours, and contract hours. All dependent variables are expressed as a percentage of the facility’s average staffing level (or hours worked) for each staff type. All regressions also contain facility and week fixed effects.

Figure 4 shows these changes by staff type, averaged across the 16 weeks after the outbreak start. The largest declines were observed for CNAs. The CNA staff size was down an average of 4.7% (95% CI, 4.0%-5.4%) of the mean staff count and 5.8% (95% CI, 5.1%-6.5%) of mean hours, while RN and LPN staff declines were considerably smaller. Rather than being because of substantially different rates of absences or departures, the main source of the difference appears to be that facilities made substantially fewer additional CNA new hires compared with RN and LPN hires.

**Figure 5.**
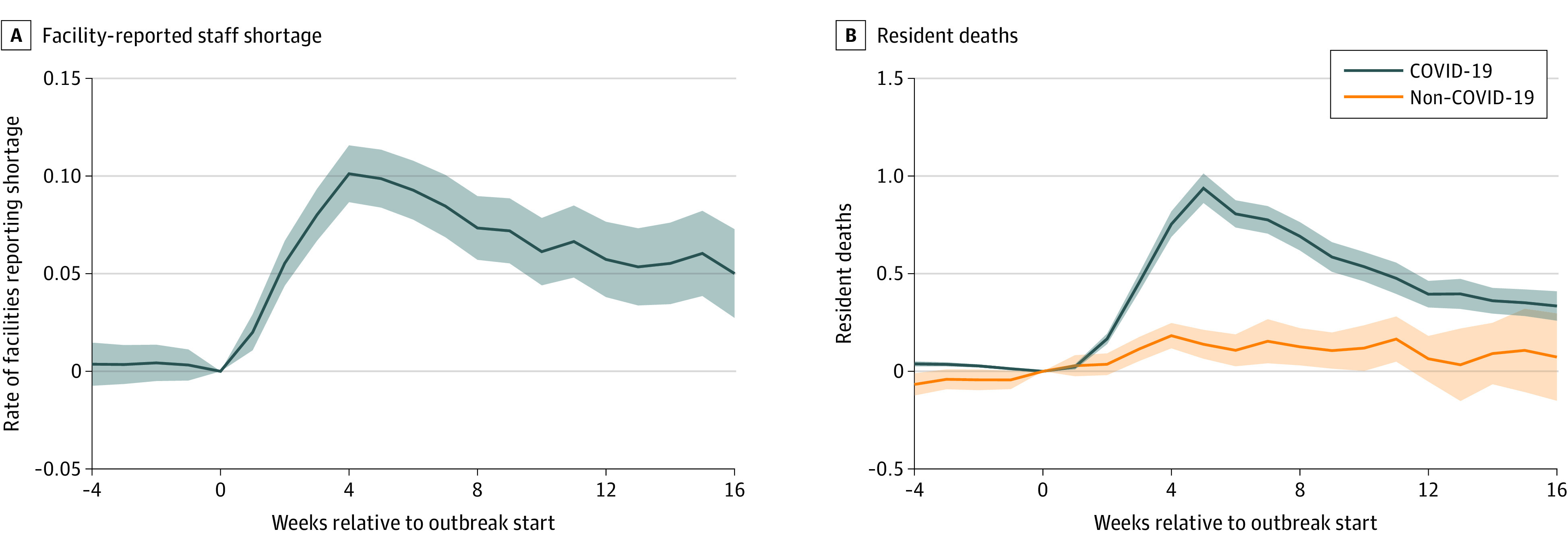
Reported Nursing Home Staff Shortages and Resident Deaths During a Severe COVID-19 Outbreak Coefficients and 95% CIs (shaded areas) are shown from facility-week regressions for an analysis sample of 2967 facilities experiencing a severe COVID-19 outbreak that started between June 14, 2020, and January 1, 2021. There are 456 029 facility-weeks between January 1, 2017, and March 31, 2021, included. Primary independent variables are event-time indicator variables relative to the outbreak start. All regressions also contain facility and week fixed effects. A, The dependent variable is an indicator for a facility reporting a shortage of nursing staff and/or aides in that week. B, The dependent variables are facility resident COVID-19–related deaths and resident non-COVID-19–related deaths in that week, as reported to the National Healthcare Safety Network.

Finally, Figure 5 explores 2 additional measures of facility stress. Figure 5A shows that outbreaks also coincided with increases in the rate of facility administrators reporting nursing staff shortages, which is 10.1 (95% CI, 8.6-11.6) percentage points higher during the fourth week of the outbreak than in other preoutbreak weeks. Figure 5B shows that resident deaths also increased during a severe outbreak. While this is primarily owing to increased deaths due to COVID-19, facilities also saw a rise in non-COVID-19–related deaths during an outbreak.

Event study coefficients for the weeks prior to an outbreak are near zero for most dependent variables. This finding supports the assumption of parallel trends that forms the basis of this article’s identification. In addition, the findings of clear shocks starting in the exact week of treatment and peaks roughly coinciding with the peak in cases offer additional validation of the identification strategy.

eFigure 3 in the Supplement shows the main findings for the other deciles of outbreaks. There were similar staffing reductions for the second decile, statistically significant reductions for the third decile, and small to no reductions for other deciles. eFigure 4 in the Supplement shows that the main findings are robust to measuring outbreak size using total cases instead of total cases per bed, suggesting that the results are not sensitive to the method of defining severe outbreaks. eFigure 5 in the Supplement shows that the main results are robust to including a control for county COVID-19 rates, suggesting that the patterns observed are specific to facility outbreaks rather than rising rates in the surrounding community. eFigure 6 in the Supplement shows that the findings are robust to using an alternative estimator from the literature that addresses negative weighting in 2-way fixed-effect event studies.

## Discussion

This study found that severe COVID-19 outbreaks were associated with statistically significantly reduced nursing staffing levels at nursing homes. Staffing reductions were driven by both temporary absences and permanent departures. Facilities were only able to partially offset these losses during outbreaks through increased new hires and the potentially costly use of contract staff and overtime. Because the use of contract hours and overtime declined after the peak outbreak weeks, staffing levels fell further after these weeks and did not recover even 16 weeks after the start of an outbreak, suggesting a lasting effect of severe outbreaks on facility staffing.

Previous work found that per-resident staffing levels were stable or higher than prepandemic levels owing to a declining resident census that outpaced reductions in staffing.^16^ We confirmed this to be true even during severe outbreaks. However, we also found that facility managers are much more likely to report staffing shortages during severe outbreaks, suggesting that per-resident staffing may not be a suitable benchmark for understanding staffing capacity during these times. The shifting composition of nursing home residents caused by declines in admissions of short-stay rehabilitative residents,^18^ and adoption of intensive infection-control practices, likely drastically altered the nature of resident care in nursing homes, potentially necessitating greater staffing per resident.

We found staffing losses were greatest for CNAs, primarily owing to a disproportionate lack of hiring to fill losses created by absences and departures. Previous work has demonstrated that CNA positions have higher rates of turnover, likely owing to their low wages and limited benefits.^6^ Similar factors may impede the filling of available CNA positions during outbreaks and raise concerns about the long-term outlook of this occupation.^19^ In contrast, RN staffing appeared to be the most stable during outbreaks, largely owing to substantial increases in new RN hires. This study is unable to distinguish whether this pattern reflects the available supply of RNs or a focused effort to bring in additional RN hours owing to their additional training and ability to implement and oversee infection control practices.

Future work is needed to determine whether these staffing reductions had adverse effects on resident quality of life, morbidity, and mortality, including by putting facilities at risk for additional outbreaks. Although descriptive, the contemporaneous increase in staffing shortages and resident non-COVID-19–related deaths is consistent with worsened resident health owing to inadequate staffing.

### Limitations

This study is limited by the fact that we are unable to observe the reasons for changes in absences, departures, and new hires. For example, we do not know whether staff absences were because of sickness and quarantine, fear of exposure, or other factors. In addition, we do not know if facilities intentionally chose to lower staffing levels owing to the occupancy declines or if facilities were unable to achieve their desired staffing because of turnover and hiring constraints. We also miss early outbreaks that were not captured by the NHSN data.^1^

## Conclusions

In light of the considerable challenges documented in this cohort study, preparations for future infectious disease outbreaks should include emergency staffing plans for nursing homes to ensure resident safety, such as centralized “strike teams” that can be temporarily deployed.^20,21^ These teams can be organized at the state or federal level and could provide supplemental staffing to facilities experiencing severe outbreaks.^22^ The American Rescue Plan Act of 2021 provided temporary funding for such teams, and anecdotal reports indicate that they were helpful in addressing emergency staff shortages.^23,24^ Policy makers might also consider broad investment in nursing home workers through better pay and benefits, such as increasing Medicaid reimbursements alongside wage pass-through requirements.^25^ Finally, policy makers should question whether traditional staffing measures accurately capture the adequacy of staffing levels during a pandemic or if new measures are needed.

## Notes

Supplement. **eAppendix.** Additional detail on data and methods **eTable.** Summary statistics of severe outbreak sample compared to full sample **eFigure 1.** Histogram of total outbreak cases per bed for all outbreaks, and definition of severe outbreak **eFigure 2.** New hires during a severe outbreak, contract and non-contract **eFigure 3.** Staffing measures during outbreaks, by outbreak decile **eFigure 4.** Staffing measures during a severe outbreak, using alternative definition of severe outbreaks (total cases) **eFigure 5.** Staffing measures during a severe outbreak, after adding additional controls of county COVID-19 prevalence **eFigure 6.** Staffing measures during a severe outbreak, estimated using Callaway Sant’Anna estimators
