# Supplementary material for: Staffing Patterns in US Nursing Homes During COVID-19 Outbreaks
Source: JAMA Health Forum. 2022 Jul 22;3(7):e222151. doi: 10.1001/jamahealthforum.2022.2151 (PMC9308062; doi:10.1001/jamahealthforum.2022.2151)
Supplement: Supplement. — eAppendix. Additional detail on data and methods eTable. Summary statistics of severe outbreak sample compared to full sample eFigure 1. Histogram of total outbreak cases per bed for all outbreaks, and definition of severe outbreak eFigure 2. New hires during a severe outbreak, contract and non-contract eFigure 3. Staffing measures during outbreaks, by outbreak decile eFigure 4. Staffing measures during a severe outbreak, using alternative definition of severe outbreaks (total cases) eFigure 5. Staffing measures during a severe outbreak, after adding additional controls of county COVID-19 prevalence eFigure 6. Staffing measures during a severe outbreak, estimated using Callaway Sant’Anna estimators [file jamahealthforum-e222151-s001.pdf]

Supplemental Online Content

Shen K, McGarry BE, Grabowski DC, Gruber J, Gandhi AD. Staffing patterns in US nursing homes during COVID-19 outbreaks. *JAMA Health Forum*. 2022;3(7):e222151. doi:10.1001/jamahealthforum.2022.2151

eAppendix. Additional detail on data and methods
eTable. Summary statistics of severe outbreak sample compared to full sample
eFigure 1. Histogram of total outbreak cases per bed for all outbreaks, and definition of severe outbreak
eFigure 2. New hires during a severe outbreak, contract and non-contract
eFigure 3. Staffing measures during outbreaks, by outbreak decile
eFigure 4. Staffing measures during a severe outbreak, using alternative definition of severe outbreaks (total cases)
eFigure 5. Staffing measures during a severe outbreak, after adding additional controls of county COVID-19 prevalence
eFigure 6. Staffing measures during a severe outbreak, estimated using Callaway Sant’Anna estimators

This supplemental material has been provided by the authors to give readers additional information about their work.

© 2022 Shen K et al. *JAMA Health Forum.*

eAppendix. Additional detail on data and methods

Staffing data and staffing measures

We first cleaned the data by keeping the last period of continuous reporting with consistent employee identifiers for each facility. We keep the last continuous period of reporting for each facility to avoid any measurement issues related to facilities not reporting data in a particular quarter (which would prevent us from being able to accurately identify new hire, absence, and departure weeks). Reporting rates in most quarters are fairly high; however, due to the COVID-19 pandemic, CMS made reporting optional for the first quarter of 2020, resulting in 19% fewer submissions than the previous quarter. Our restriction means that for these facilities, we keep only the data starting in Q2 2020. We assume there has been a change in the assignment of employee identifiers as facilities where more than 75% of the present staff in a given week is newly hired, and the overall staff size has not changed by more than 25%. In this case, we again keep only the period following the change in employee identifiers, so that we are able to measure new hires, absences, and departures.

To avoid issues related to left- or right-censoring of the data at the beginning and end of the available data, we construct our new hire, absence, and departure measures using a 12-week lookback and a 12-week follow-up period. Thus, our final dataset contains these measures from April 1, 2017 through March 31, 2021 (12 weeks after the start of the available data to 12 weeks before the end of the available data). Specifically, we define new hires as someone who is working for the first time in 12 weeks, departures as someone who is working their last week for the next 12 weeks, and absences as people who did not work in a given week, but will return within 12 weeks from the last week in which they worked.

Case data and outbreak definition

We use data from the National Healthcare Safety Network (NHSN) COVID-19 Nursing Home Database to identify large outbreaks. This data consists of weekly reports of facility COVID-19 cases and deaths for residents and staff. The first data submission was on May 24, 2020, meaning that we are not able to identify outbreaks in the first two months of the pandemic. We also exclude the first submission of data because for that week, facilities had the option of either reporting retrospective data on cases and deaths from COVID-19 dating back to January 1, 2020, or simply reporting new cases and deaths from the past week. We thus have new weekly cases and deaths beginning with the week of May 31, 2020.

We define an outbreak as any period of consecutive weeks of positive COVID-19 (staff or resident) cases that is preceded and followed by at least two weeks with no new cases. This means that two periods of cases that are separated by only one week with no new cases are counted as the same outbreak, rather than two different outbreaks. We define the outbreak's start as the first week of cases within such a period. Because our analysis is indexed by the start of the outbreak, we exclude any outbreaks that were ongoing at the beginning of the available data (May 31, 2020). Thus the earliest outbreak start date in our analysis is June 14, 2020 (two weeks after the start of the data). We consider all outbreaks that started between this date and January 1, 2021.

© 2022 Shen K et al. *JAMA Health Forum.*

For each of these outbreaks, we compute the total number of resident and staff cases at that facility during the outbreak. We label the top decile of these as severe outbreaks, and use this as our exposure measure.

Analysis sample definition

Using the NHSN data, we identify 39,485 outbreaks in 15,171 facilities. We restrict our analysis to the top decile of these outbreaks in terms of total cases per bed, corresponding to 3,939 outbreaks for 3,875 facilities. Of these, we keep only the first outbreak for each facility (dropping N=64 outbreaks) and also drop N=908 outbreaks for which we do not observe the facility in the PBJ staffing data in the week of the outbreak start. This results in a final analysis sample of N=2,967 outbreaks and N=456,029 facility-weeks between January 1, 2017 and March 31, 2021.

© 2022 Shen K et al. *JAMA Health Forum.*

eTable. Summary statistics of severe outbreak sample compared to full sample

	All Outbreaks, Mean (SD)	Severe Outbreaks, Mean (SD)	Analysis Sample, Mean (SD)	Alternative Def. Analysis Sample, Mean (SD)
Number of outbreaks	39,485	3,939	2,967	3,010
Number of unique facilities	15,171	3,875	2,967	3,010
Facilities matched to PBJ	13,257	2,995	2,967	3,010
Pre-outbreak weeks	3,477,858	375,821	375,077	377,449
Post-outbreak weeks	835,198	80,516	79,085	80,952
**Outbreak Characteristics**				
Cases Per Bed	0.30 (0.51)	1.49 (0.74)	1.47 (0.69)	1.29 (0.76)
Total Outbreak Cases	29.6 (53.2)	135.1 (89.2)	134.7 (87.6)	153.8 (76.3)
Outbreak Start Date	Sept 21, 2020 (57 days)	Sept 26, 2020 (54 days)	Sept 27, 2020 (53 days)	Sept 26, 2020 (53 days)
Outbreak Length (weeks)	5.3 (5.9)	14.5 (8.2)	14.4 (8.3)	16.0 (8.0)
**Facility Characteristics**				
Beds	106 (61)	93 (44)	96 (44)	134 (66)
For-profit	0.70 (0.46)	0.71 (0.46)	0.72 (0.45)	0.76 (0.42)
Star Rating	3.1 (1.4)	3.2 (1.4)	3.3 (1.4)	3.0 (1.4)
Region				
Northeast	0.17 (0.37)	0.12 (0.31)	0.12 (0.33)	0.17 (0.37)
Midwest	0.34 (0.47)	0.36 (0.48)	0.37 (0.48)	0.27 (0.45)
South	0.36 (0.48)	0.33 (0.47)	0.31 (0.46)	0.38 (0.49)
West	0.16 (0.36)	0.19 (0.39)	0.40 (0.39)	0.18 (0.38)
**Pre-Outbreak Weekly Measures**				
Resident Census	82 (46)	79 (38)	79 (39)	109 (52)
Nursing Staff Count	67 (37)	67 (33)	69 (33)	88 (46)
RN	11 (8.0)	11 (8.1)	11 (8.0)	14 (11)
LPN	15 (10)	15 (9.4)	15 (9.4)	21 (12)
CNA	41 (24)	41 (21)	41 (21)	54 (29)
Nursing Staff Hours	2191 (1239)	2148 (1091)	2145 (1112)	2894 (1529)
RN	353 (261)	344 (267)	344 (263)	448 (349)
LPN	517 (343)	506 (321)	506 (321)	700 (399)
CNA	1320 (781)	2148 (1112)	1295 (694)	1747 (971)

Notes: Average outbreak, facility, and pre-outbreak weekly measures for full sample of outbreaks (column 1), severe outbreaks (column 2), analysis sample of severe outbreaks which is

© 2022 Shen K et al. *JAMA Health Forum.*

used in the main text (column 3), and alternative analysis sample of severe outbreaks, defined using total cases rather than total cases per bed (column 4). The difference between column 2 and column 3 represents outbreaks dropped due to not matching to the PBJ data and keeping the first severe outbreak for each facility.

© 2022 Shen K et al. *JAMA Health Forum.*

eFigure 1. Histogram of total outbreak cases per bed for all outbreaks, and definition of severe outbreak

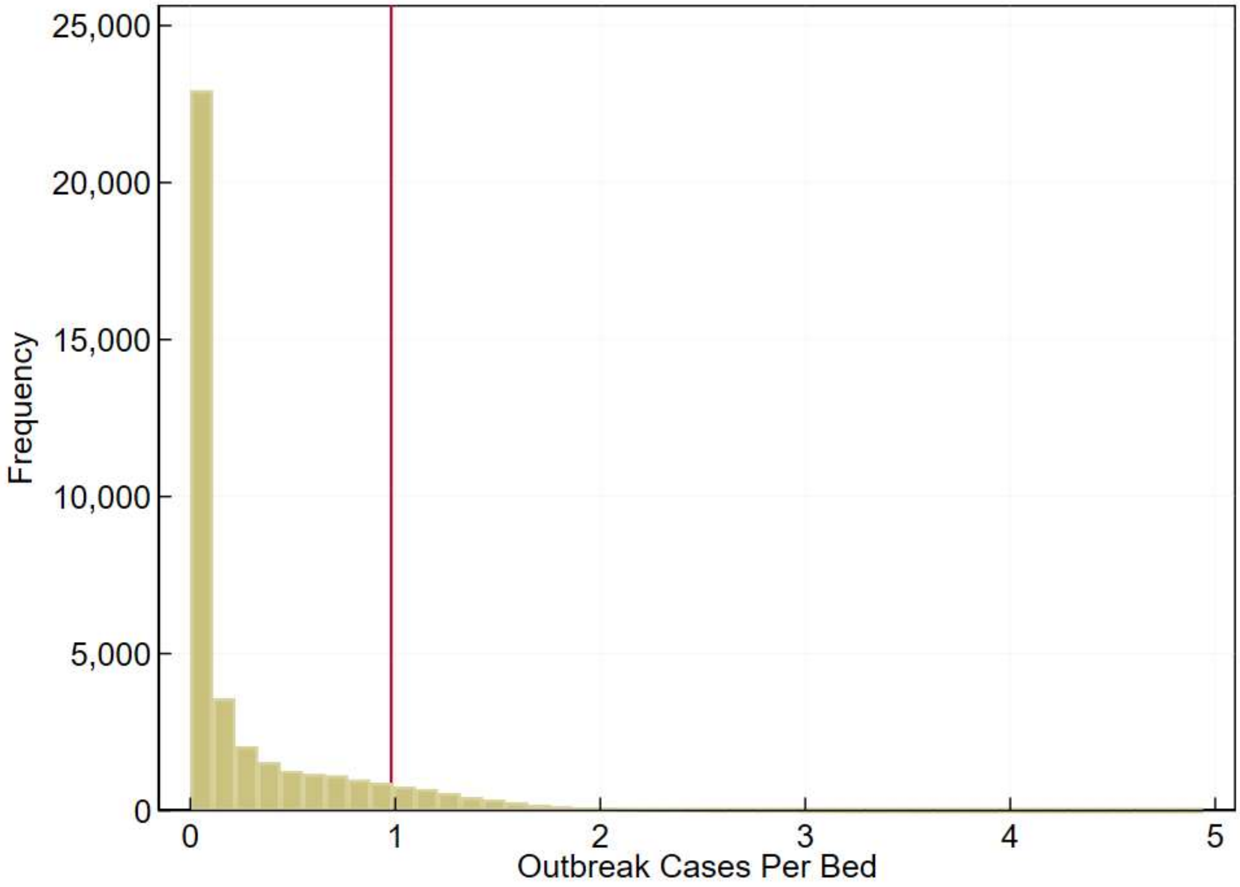

Notes: This histogram calculates total outbreak cases per bed for N=39,485 outbreaks using the NHSN data. The red line indicates the top decile of these outbreaks, which corresponds to the definition of a "severe outbreak" in the text (outbreaks that exceeded 0.98 total resident and staff cases per bed). We dropped 30 outliers with more than 5 cases per bed from this graph for ease of presentation, although they are included in the analysis sample.

© 2022 Shen K et al. *JAMA Health Forum.*

eFigure 2. New hires during a severe outbreak, contract and non-contract

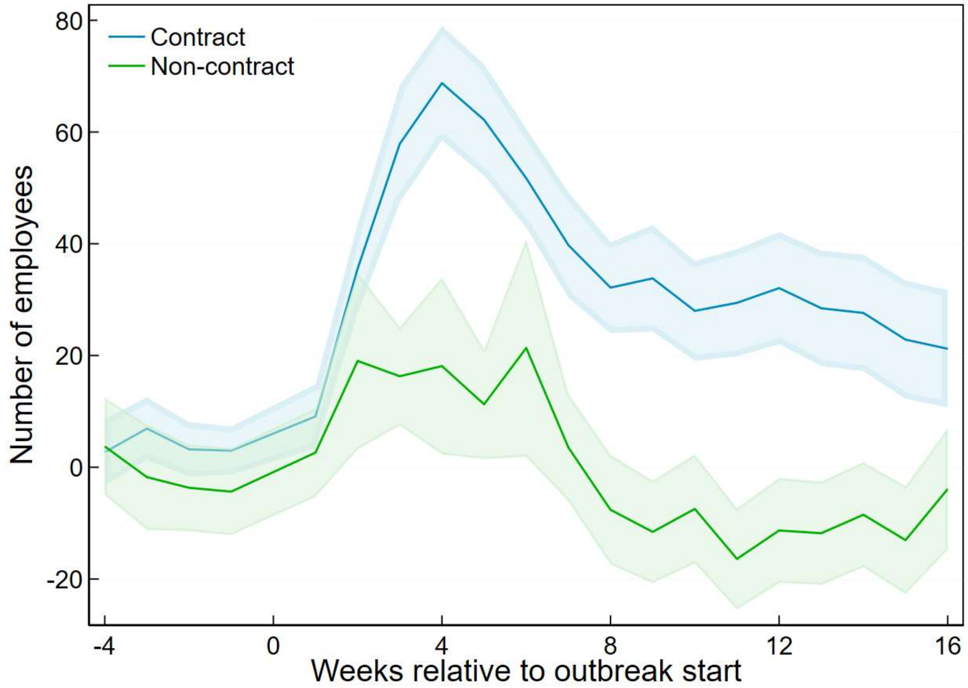

Notes: Coefficients and 95% confidence intervals from facility-week regressions for all facilities experiencing a severe outbreak in the analysis sample from 2017-2020. Primary independent variables are event-time indicator variables relative to the outbreak start. Coefficients for weeks -4 to 16 are shown. All regressions also contain facility and week fixed effects. Dependent variables are (a) the number of new contract nursing staff members in a given week, (b) the number of new non-contract nursing staff members in a given week.

© 2022 Shen K et al. *JAMA Health Forum.*

eFigure 3. Staffing measures during outbreaks, by outbreak decile

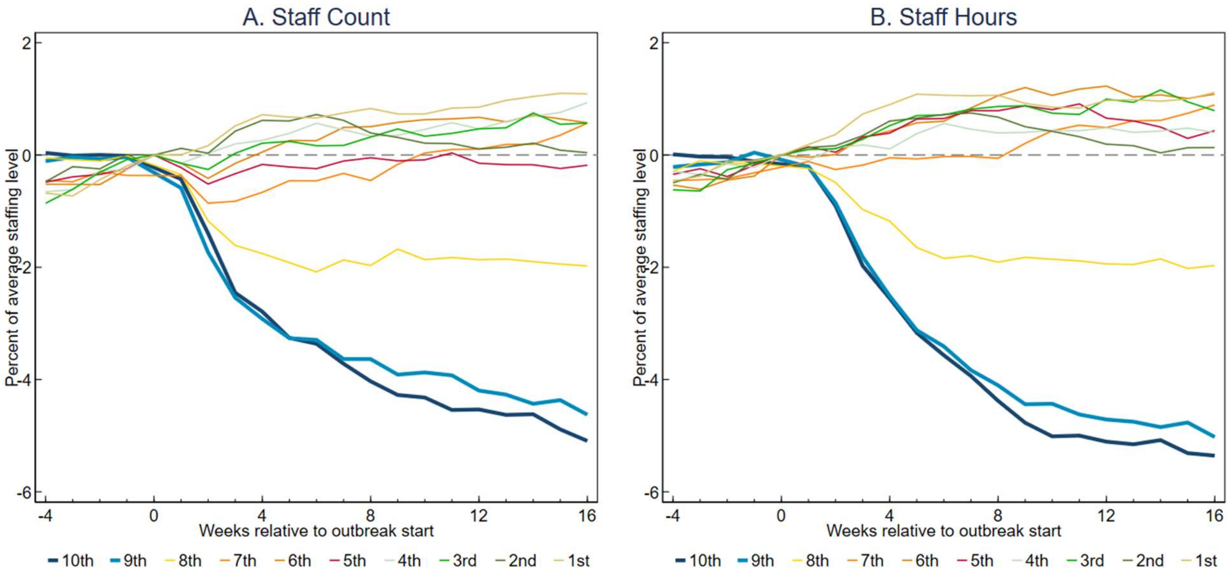

Notes: Coefficients and 95% confidence intervals from facility-week regressions for all facilities experiencing any outbreak in the analysis sample from 2017-2020. Facilities may be included in the sample multiple times, once per outbreak. Outbreaks are sorted into deciles by their “severity,” defined as total outbreak cases per bed. Primary independent variables are event-time indicator variables relative to the outbreak start. Coefficients for weeks -4 to 16 are shown. All regressions also contain facility and week fixed effects. Dependent variables are (a) the number of unique nursing staff members who worked in a given week, (b) total hours worked by nursing staff in a given week.

© 2022 Shen K et al. *JAMA Health Forum.*

eFigure 4. Staffing measures during a severe outbreak, using alternative definition of severe outbreaks (total cases)

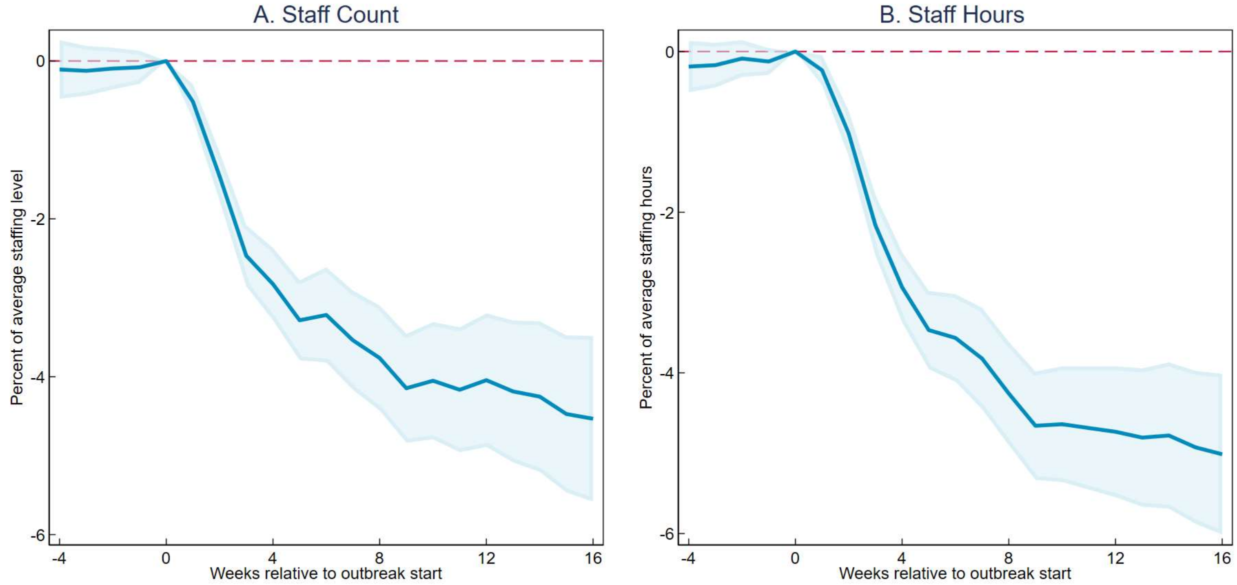

Notes: Coefficients and 95% confidence intervals from facility-week regressions for all facilities experiencing a severe outbreak in the analysis sample from 2017-2020. Primary independent variables are event-time indicator variables relative to the outbreak start. All regressions also contain facility and week fixed effects. Dependent variables are (a) the number of unique nursing staff members who worked in a given week, (b) total hours worked by nursing staff in a given week.

eFigure 5. Staffing measures during a severe outbreak, after adding additional controls of county COVID-19 prevalence

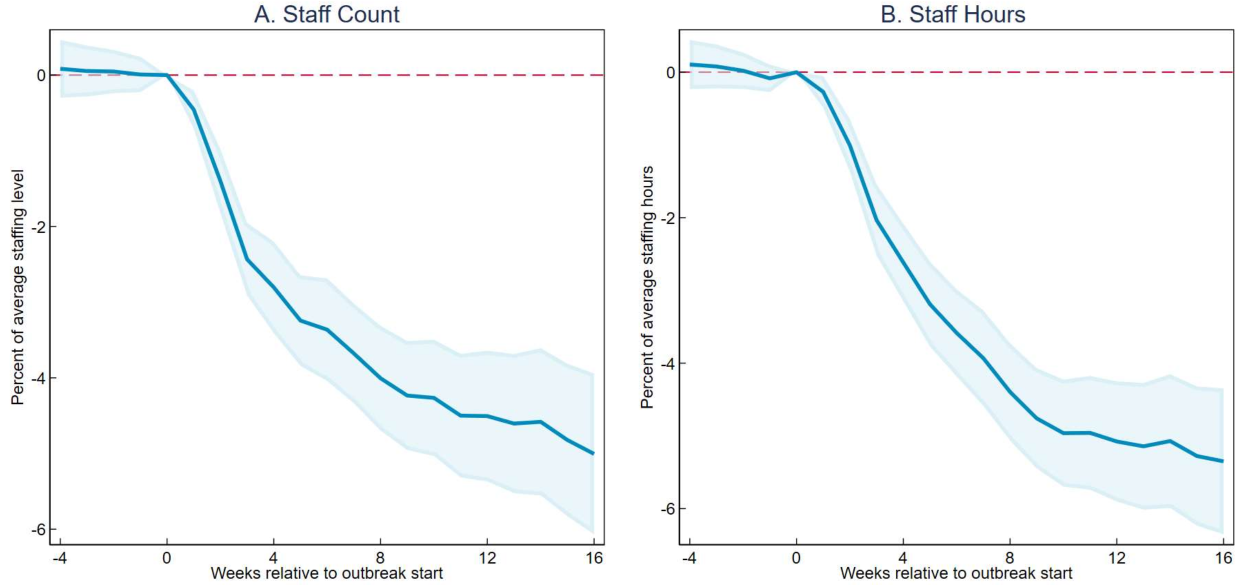

Notes: Coefficients and 95% confidence intervals from facility-week regressions for all facilities experiencing a severe outbreak in the analysis sample from 2017-2020. Primary independent variables are event-time indicator variables relative to the outbreak start. All regressions also contain facility and week fixed effects, as well as the weekly county case rate for the facility's county. Dependent variables are (a) the number of unique nursing staff members who worked in a given week, (b) total hours worked by nursing staff in a given week.

© 2022 Shen K et al. *JAMA Health Forum.*

eFigure 6. Staffing measures during a severe outbreak, estimated using Callaway Sant'Anna estimators

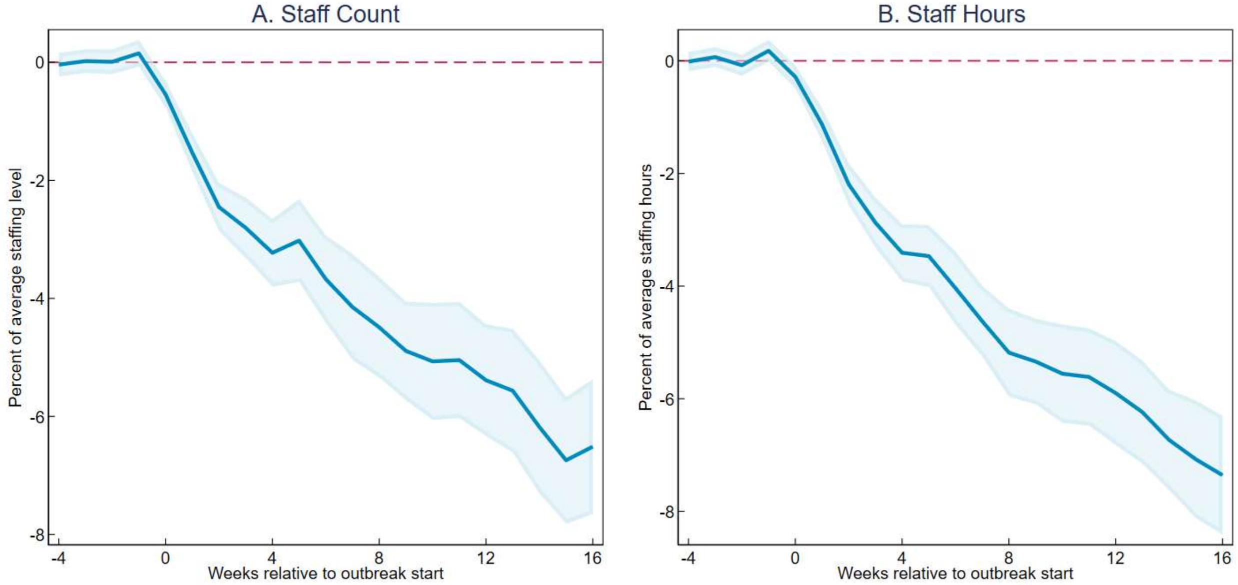

Notes: Coefficients and 95% confidence intervals represent event-time average treatment effects using the average group-time treatment effect estimators from Callaway and Sant'Anna (2021). Sample is all facilities experiencing a severe outbreak in our data from 2017-2020. Control facilities are not-yet treated and an unbalanced panel is used. Primary independent variables are event-time indicator variables relative to the outbreak start. All regressions also contain facility and week fixed effects. Dependent variables are (a) the number of unique nursing staff members who worked in a given week, (b) total hours worked by nursing staff in a given week.

© 2022 Shen K et al. *JAMA Health Forum.*
